# Prehospital epinephrine administration and survival among patients with unshockable initial rhythm after out-of-hospital cardiac arrest

**DOI:** 10.1186/cc12240

**Published:** 2013-03-19

**Authors:** Y Goto, T Maeda, Y Goto

**Affiliations:** 1 Kanazawa University Hospital, Kanazawa, Japan; 2 Yawata Medical Center, Komatsu, Japan

## Introduction

Epinephrine has been a cornerstone of cardiac resuscitation and advanced cardiac life support since the 1960s. However, there is little evidence from clinical trials that epinephrine administration after out-of-hospital cardiac arrest (OHCA) improves long-term survival. There would be subsets of patients for whom epinephrine administration is in fact beneficial. Our objective was to determine whether prehospital epinephrine administration would improve survival at 1 month in OHCA patients with unshockable initial rhythm.

## Methods

We analyzed data for 383,045 adult OHCA patients with unshockable initial rhythm, from a prospectively recorded nationwide Utstein-style Japanese database for 2007 to 2010. We divided these patients into two cohorts: prehospital epinephrine administration cohort (*n *= 30,237) and non-epinephrine administration cohort (*n *= 352,808). The endpoints were 1-month survival after OHCA, prehospital return of spontaneous circulations (ROSCs), and 1-month survival with favorable neurological outcome (Cerebral Performance Category (CPC) scale, categories 1 to 2) at 1 month.

## Results

The rate of 1-month survival was 3.72% for the epinephrine administration cohort and 2.49% for the non-epinephrine administration cohort, 17.9% versus 3.0% for prehospital ROSC, and 0.57% versus 0.77% for CPC 1 to 2 (all *P <*0.0001). Positive associations were observed between epinephrine administration and 1-month survival (adjusted odds ratio (aOR), 1.18; 95% CI, 1.11 to 1.27), and prehospital ROSC (aOR, 5.50; 95% CI, 5.29 to 5.72; all *P <*0.0001). Negative association was observed between epinephrine administration and CPC 1 to 2 (aOR, 0.56; 95% CI, 0.48 to 0.66; *P <*0.0001). Multivariate logistic analysis revealed that age (<66 years; aOR, 4.31; 95% CI, 2.47 to 8.01), total dose of epinephrine (1 mg; aOR, 3.65; 95% CI, 2.61 to 5.18), call-response time (<5 minutes; aOR, 3.58; 95% CI, 1.98 to 6.69), witnessed arrest (aOR, 2.17; 95% CI, 1.51 to 3.16), and pulseless electrical activity as an initial rhythm (aOR, 2.02; 95% CI, 1.46 to 2.80) were significantly associated with CPC 1 to 2 at 1 month in the epinephrine administration cohort.

## Conclusion

In OHCA patients with unshockable initial rhythm, prehospital epinephrine administration significantly increased the rate of survival at 1 month after cardiac arrest. The best single predictor for favorable neurological outcomes at 1 month following prehospital epinephrine administration after cardiac arrest was age (<66 years) followed by total dose of epinephrine (1 mg) and then by call-response time (<5 minutes).

